# Effect of Microstructure and Compressive Residual Stress on the Fatigue Performance of AISI 4140 Steel with QPQ Salt-Bath Nitro-Carburizing

**DOI:** 10.3390/ma18091995

**Published:** 2025-04-28

**Authors:** Hao Chen, Tai-Cheng Chen, Hsiao-Hung Hsu, Leu-Wen Tsay

**Affiliations:** 1 Department of Optoelectronics and Materials Technology, National Taiwan Ocean University, Keelung 202301, Taiwan; howard102697@gmail.com; 2 Department of Material Research, National Atomic Research Institute, Taoyuan 325207, Taiwan; tcchen@nari.org.tw; 3 Iron and Steel Research and Development Department, China Steel Corporation, Kaohsiung 812401, Taiwan; 175877@mail.csc.com.tw; 4 Vincent Vacuum-Tech Co., Ltd., Taoyuan 326019, Taiwan

**Keywords:** AISI 4140, micro-shot peening, salt-bath nitrocarburizing, post-oxidation, rotating bending fatigue

## Abstract

Quench–polish–quench (QPQ) nitro-carburizing of AISI 4140 steel in a salt bath was performed in this study. Nitro-carburizing in a salt bath enhanced the formation of Fe-nitride on the outer surface layer. Moreover, the oxidizing treatment formed a thin oxide layer decorated on the outermost part of the QPQ-treated sample. The dense compound layer formed after nitro-carburizing in a salt bath consisted of refined granular Fe_3_N and transformed to Fe_2_N after post-oxidation treatment. Micro-shot peening (MSP) was adopted before QPQ treatment to increase the treated steel’s fatigue performance. The results indicated that MSP slightly increased the thickness of the compound layer and harden depth, but it had little effect on improving the fatigue strength/life of the QPQ-treated sample (SP-QPQ) compared to the non-peened one (NP-QPQ). A deep compressive residual stress (CRS) field (about 200 μm) and a hard nitrided layer showed a noticeable improvement in the fatigue performance of the QPQ-treated ones relative to the 4140 substrates tempered at 570 °C. The ease of slipping or deforming on the substrate surface was responsible for its poor resistance to fatigue failure. The cracking and spalling of the brittle surface layer were the causes for the fatigue crack initiation and growth of all of the QPQ-treated samples fatigue-loaded at/above 875 MPa. It was noticed that fatigue crack initiation at the subsurface inclusions was more likely to occur in the SP-QPQ sample fatigue-loading at 850 MPa or slightly above the fatigue limit.

## 1. Introduction

Various nitriding processes [1,2,3,4,5] are applied to modify the surface properties of critical components for different industries, including low alloy steel, mold and tool steels, and stainless steels. The quench–polish–quench (QPQ) process, a complex salt-bath process, is developed initially to improve the fatigue, wear, and corrosion resistance of automotive items [6,7,8,9,10,11,12] and extends its usage to austenitic stainless steel afterward [13,14]. After sequential polishing and oxidation after nitro-carburizing, the QPQ-treated components show a black-shinning and smooth surface feature. The QPQ-treated steel generally comprises three layers: an oxide film on the outer surface, a compound layer beneath the oxide film, and a diffusion zone adjacent to the substrate. The oxide film inherently has high corrosion resistance; the compound layer provides high wear resistance, and the diffusion zone increases fatigue strength [6,8,10,12]. To reduce the harmful effect of cyanides or cyanates, non-toxic salt has been developed recently to nitride the 0.2%C steel [15].

Shot-peening introduces high residual stress in compression [16], which can enhance the lifetime and increase the fatigue strength of the peened components [17,18,19]. Fine particles or micro-shots are applied to the peening process to increase fatigue strength and reduce surface roughness after air-blast peening [20,21,22,23]. Nitriding is also known to improve the fatigue performance of the 4140 steel [24,25]. Increasing the compound layer thickness of the nitrided 4135 steel is reported to increase the compressive residual stress (CRS) depth, increasing its notched fatigue strength [26]. The nitrided 4135 steel pre-peened with fine particles has a higher surface hardness and a lower friction coefficient than the unpeened nitrided one, which is owing to the presence of a thick compound layer without defects [27]. It is reported that either shot-peening before nitriding [28] or peening after nitriding [29] can improve the fatigue strength of nitrided 4140 steel. Moreover, the fatigue crack initiation site tends to move from the external surface to the subsurface zone in gas-nitrided 4140 steel in a high-cycle fatigue regime [24].

Salt-bath nitriding is reported to significantly improve the fatigue performance of 13Cr-4Ni martensitic stainless steel [30]. Post-oxidation after salt-bath nitro-carburizing can further increase the corrosion resistance of the treated components [8]. The corrosion resistance of nitro-carburized 4135 steel can be significantly improved after post-oxidation at 400 °C for less than 2 h [31]. Furthermore, pre-oxidation in a salt bath at 350 °C before nitro-carburizing 1045 steel assists the growth of Fe_3_O_4_, increasing the efficiency of salt-bath nitriding [32]. Moreover, the oxide layer has no adverse effect on its fatigue strength for the salt-bath-nitrided and post-oxidized medium C steel [33]. In the case of the 4135 steel nitro-carburized and post-oxidized in a salt bath, subsurface fatigue cracks are initiated in a short fatigue life regime, and this changes to internal crack initiation at inclusion in a longer fatigue life regime [34]. Even in 3.5 wt% NaCl solution, the nitro-carburized and post-oxidized 4330V steel shows a much better corrosion fatigue performance than the untreated one [35].

Heat-treatable low alloy steel, like AISI 4140 steel, is used for the shaft, piston tube, seat post, and fork tube after quenching and tempering. To further enhance the wear and corrosion resistance, QPQ salt-bath treatment of the 4140 steel components is performed. In this study, micro-shot peening (MSP) is carried out as a pre-treatment before the QPQ process to introduce RCS and refined grains in the treated 4140 steel [28] compared to the non-peened ones. The microstructure and the fracture feature of the investigated sample were inspected by a scanning electron microscope (SEM). The delicate microstructure of the nitrided layer was investigated by electron backscatter diffraction (EBSD). Rotating bending fatigue tests were performed to evaluate the fatigue performance of the analyzed samples and compared to the 4140 substrates after tempering at 570 °C for 2 h.

## 2. Material and Experimental Procedures

### 2.1. Preparation of the Sample

**Figure 1:**
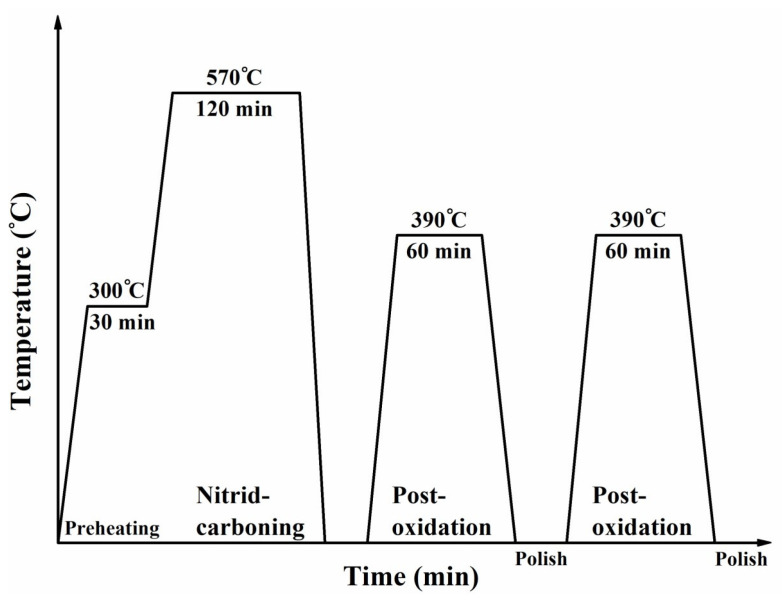
Schematic diagram showing the details of salt-bath nitrocarburizing and post-oxidation, also known as quench–polish–quench (QPQ), processes used in this work.

**Figure 2:**
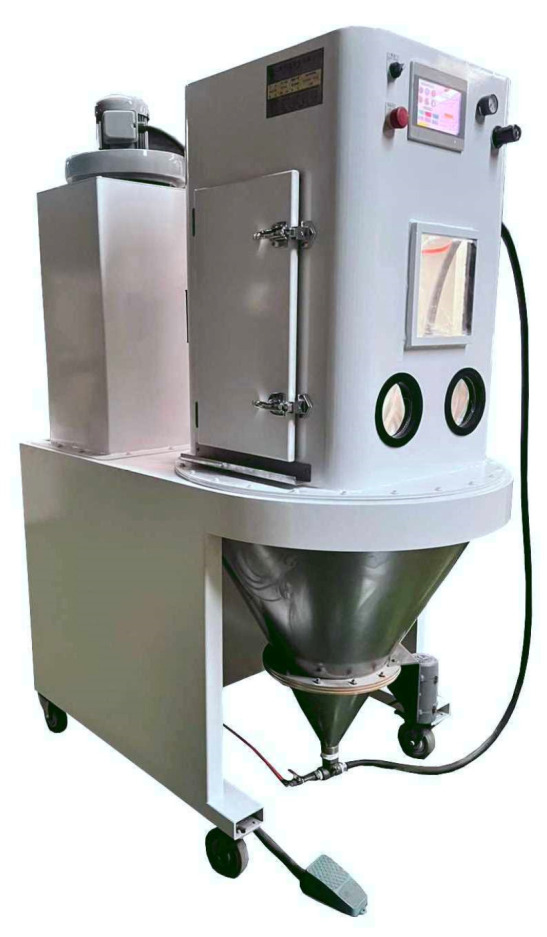
Equipment used for micro-shot peening.

An AISI 4140 steel bar of 12 mm diameter was provided by China Steel Corporation in Taiwan. The 4140 composition in weight percent (wt%) was 0.42 C, 0.81 Mn, 0.011 P, 0.006 S, 0.23 Si, 1.01 Cr, 0.17 Mo, 0.01 Ni, and balanced Fe. The steel bar was austenized at 865 °C/0.5 h and then subjected to oil quenching, followed by tempering at 200 °C/0.5 h; the sample had a hardness of approximately 500HV_0.3_ and was labeled as QT200. Figure 1 displays the thermo-chemical treatment of the QPQ process. Salt-bath nitro-carburizing was conducted at 570 °C for 2 h, followed by water-quenching. The nitro-carburized samples were treated with the first cycle of post-oxidation at 390 °C for 1 h. The oxidized sample was polished to achieve a smooth surface. Then, a secondary cycle of post-oxidation and polishing was carried out to improve the corrosion resistance and reduce the surface roughness of the treated samples. Micro-shot peening [28] was performed on the 200 °C tempered 4140 steel before QPQ treatment. Figure 2 displays the appearance of the customized equipment for micro-shot peening (MSP), which was manufactuered by Rich Sou Technology Co., Ltd. (Kaohsiung, Taiwan). The shot balls were accelerated under constant pressure and the shot balls were feeding at a constant rate to bombard the samples. The nitro-carburized sample with MSP was designated as the SP-QPQ sample, in contrast to the non-peened (NP-QPQ) one. To evaluate the effect of nitro-carburizing on increasing the fatigue properties of 4140 steel, 4140 steel tempered at 570 °C for 2 h was used and labeled as the QT570 specimen.

### 2.2. Measurement of Hardness and Fatigue Testing

**Figure 3:**
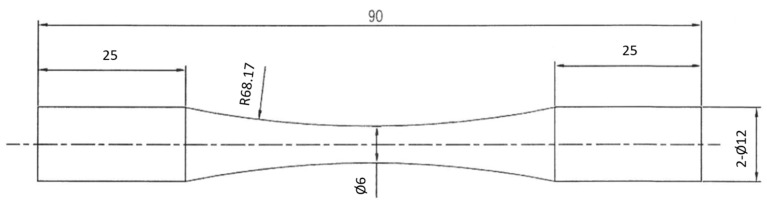
Schematic diagram showing the dimensions of the fatigue sample (unit: mm).

An MVK-G1500 Vickers tester (Mitutoyo, Kawasaki, Japan) was used to measure the micro-hardness profile of the investigated samples. The surface metrology of the inspected samples with and without MSP was investigated using a Contour GT-K 3D optical profiler (Bruker, Billerica, MA, USA). The surface roughness of the examined sample was quntified by Sa (arithmetical mean height of the surface), Sp (maximum peak height of the surface), and Sv (maximum pit depth of the surface). Figure 3 is the schematic diagram showing the dimensions of the rotating beam fatigue specimen used in this work. Fatigue tests were conducted using a rotating bending fatigue instrument, manufactured by Chun Yen Testing Machines Co., Ltd. (Taichung, Taiwan), operating at a frequency of 1500 cycles/min at room temperature. The stress amplitude (S) versus cycle number (N) curves were recorded with three specimens under the specific fatigue-loading.

### 2.3. Microstructural and Fracture Feature Observation

A D2 Phaser X-ray diffractometer (XRD, Bruker, Billerica, MA, USA) utilizing Cu Kα radiation was employed to identify the phase constituents of the nitride layer for investigated samples. The microstructures in the cross-sectional view and the examined sample’s fatigue-fractured appearance were inspected using an S-3400N SEM (Hitachi, Tokyo, Japan). The chemical composition at different sites of the QPQ-treated sample was analyzed with a JXA-8200 electron probe micro-analyzer (EPMA, JEOL, Tokyo, Japan) equipped with a wavelength-dispersive spectroscope (WDS). To identify distinct phases around the nitrided layer, the QPQ-treated samples were analyzed by using a JSM-7100F SEM (Oxford Instruments, Abingdon, UK) equipped with a NordlysMax^2^ electron backscatter diffraction (EBSD, Oxford Instruments, Abingdon, UK) detector.

### 2.4. Measurement of Residual Stress

A µ-X360s residual stress analyzer (Pulstec, Hamamatsu, Japan) [36] employing Cr Kα radiation was used to collect all of the diffracted beams from the irradiated surface of the inspected sample with numerous grains in distinct orientations. With the internal stress, the Debye ring related to the specific lattice plane of the examined sample can be distorted. With the aid of the developed software (version 3.2.7), the Debye ring was cut into 500 segments. The diffraction curves associated with distinct segments were superimposed after the diffraction test [36]. The full width at half maximum (FWHM) peak of the (103)_Fe2N_ (2θ, 135.5°) and (211)_α_ (2θ, 156.4°) Debye ring was utilized to qualitatively assess the lattice distortion of the nitrided sample. The difference in the 2θ angle of the Debye ring of the nitrided sample and referred phase’s angle was used to calculate the residual stress field of the nitrided sample. The residual stress of the nitrided sample was measured by using the cosα method [37,38]. To measure the residual stress at a specific depth of the sample, the material was removed gradually using an electrochemical polisher. After removing the material layer by layer, the residual stress profile in the thickness direction of the tested samples was determined.

## 3. Results

### 3.1. Surface Texture

**Figure 4:**
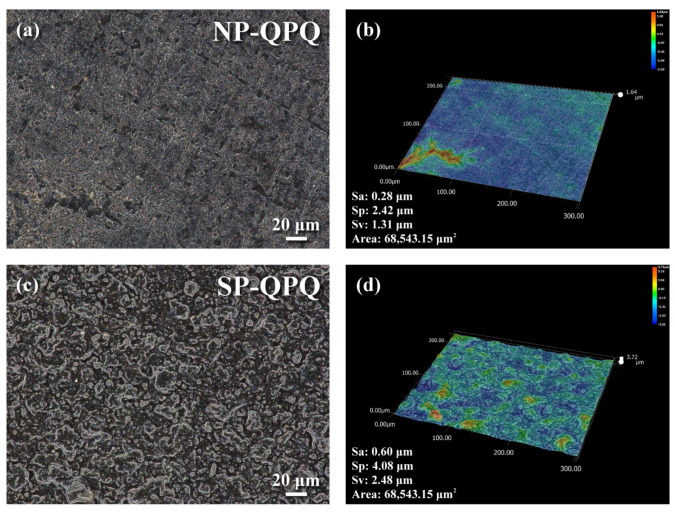
(**a**,**c**) Surface morphology and (**b**,**d**) 3D contour profile of the investigated samples afetr QPQ treatment. (**a**,**b**) The non-peened and (**c**,**d**) shot-peened samples.

Figure 4 displays the SEM morphology of the NP-QPQ and SP-QPQ samples, and the optical profiler inspected the surface topography and roughness. The 4140 steel, which was solutionized and tempered at 200 °C/0.5 h (QT200), was ground with 1000-grit SiC paper before subsequent treatment. The surface roughness of the investigated samples in distinct conditions is listed in Table 1. SEM micrographs showed that the NP sample after QPQ treatment displayed the grounded traces (Figure 4a), as compared with the appearance of fine dents in the SP-QPQ-treated one (Figure 4c). The surface roughness of the ground 4140 steel [28] was Sa: 0.31 μm, Sp: 2.02 μm, and Sv: 1.75 μm. After peening the ground 4140 steel, the Sa, Sp, and Sv of the as-peened 4140 increased to 0.50, 2.46, and 2.35 μm [28], respectively. As listed in Table 1, the SP-QPQ sample displayed a little higher surface roughness than the NP-QPQ one; in addition, the NP-QPQ sample showed a little smooth texture relative to the SP-QPQ one. The surface topography of the NP-QPQ specimen, shown in Figure 4b, exhibited a very smooth surface with some aligned and accumulated pits. The exact reasons for such events were not known at this moment, which could be partly due to the initially deep grooves after grinding. By contrast, the SP-QPQ specimen, as shown in Figure 4d, displayed the dimple feature caused by MSP and was responsible for the increased surface roughness.

### 3.2. Microstructures of the QPQ-Treated Sample

**Figure 5:**
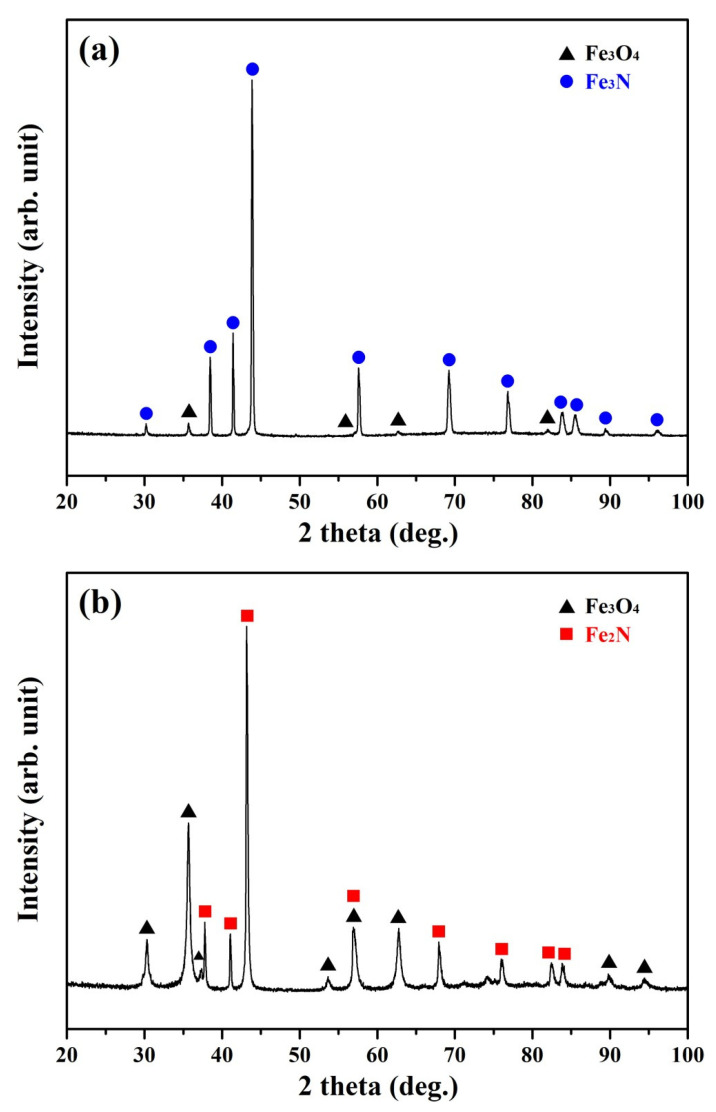
XRD spectrum detected from the outer surface of the non-peened sample in the (**a**) nitro-carburized and (**b**) post-oxidized conditions.

The phase constituents of the QPQ-treated samples analyzed by XRD are displayed in Figure 5. The XRD pattern demonstrated that Fe_3_N mixed with a small amount of Fe_3_O_4_ was found around the surface zone of the treated sample after salt-bath nitrocarburizing (Figure 5a). Similarly, iron-borides are formed on the exterior surface of the pipe line steel after packed powder boriding to increase its surface hardness, Young’s modulus, and erosion–corrosion resistance [39,40]. The boride layer thickness is reported to increase with increasing treating time and temperature [39]. Because the salt-bath nitriding temperature was as low as 570 °C, grain coarsening did not happen after nitriding for two hrs. By contrast, the temperature of packed powder boriding can be as high as 1000 °C [39]. Therefore, a coarse-grained structure can be formed in the borided sample treated at such elevated temperatures. The formation of a small amount of Fe_3_O_4_ was attributed to the air-oxidation of the nitro-carburized sample at the elevated temperature after removing the nitro-carburized sample from the salt bath. After post-oxidation, Fe_2_N replaced Fe_3_N along with the increased amount of Fe_3_O_4_ formed on the sample surface. Based on the result shown in Figure 5b, the superficial oxide layer formed on the outer surface of the QPQ-treated sample was anticipated to increase its corrosion resistance.

**Figure 6:**
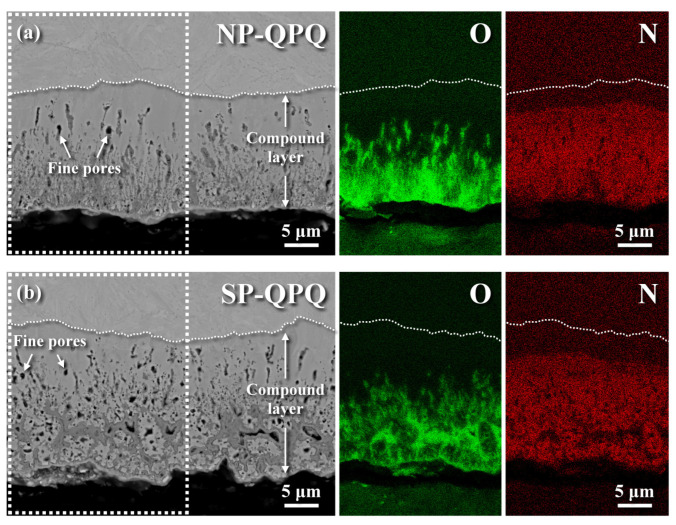
SEM micrographs of the examined samples after QPQ treatment in a cross-sectional view: (**a**) the microstructure of the NP-QPQ sampleand (**b**) the microstructure of the SP-QPQ sample. The O and N mapping of the QPQ-treated sample determined by EDS analysis of the dashed zone are shown in (**a**,**b**).

Figure 6 shows the SEM microstructures of the QPQ-treated samples in a cross-sectional view with or without shot-peening. Both QPQ-treated samples consisted of a compound layer with a few delicate pores in the compound layer (Figure 6a,b). It was noticed that the outermost profile of the SP-QPQ sample (Figure 6b) displayed a more tortuous surface profile relative to the NP-QPQ one (Figure 6a), which was associated with the slightly higher surface roughness after MSP. Moreover, network grey zones were found near the outer surface layer of the SP-QPQ sample, whereas a streak-like grey phase was more likely to be observed in the lower portion of the compound layer of the NP-QPQ specimen. Moreover, the compound layer of the NP-QPQ specimen had a more significant portion of a streak-like grey phase than that of the SP-QPQ one. The N and O maps analyzed by X-Max^N^ energy-dispersive X-ray spectroscopy (EDS, Oxford Instruments, Abingdon, UK) were made to show the elemental distributions within the dashed zone of the QPQ-treated specimen, as shown in Figure 6a,b. Overall, N was rich in the front portion of the compound layer and showed a sharp decrease in its content in the zone ahead of the interface between the compound layer and substrate. Based on the N map, the pore and grey phase in the compound layer lacked N relative to the nitride layer. By contrast, O was more likely to concentrate in the surface layer of about 5 μm in thickness and diffuse inwards. The grey zone was rich in O and could be related to the formation of iron oxide (Fe_3_O_4_), which was confirmed by XRD shown in Figure 5. It was deduced that the oxide film coating the surface of the QPQ-treated specimen was beneficial for increasing the corrosion resistance of the QPQ-treated sample. Meanwhile, the strike-like oxide was also associated with the O diffusion path.

Table 2 lists the chemical compositions in distinct zones of the SP-QPQ-treated sample analyzed by EPMA. The results revealed that the N content could be as high as 5.45 wt% in the near-surface compound layer and decrease abruptly to 0.55 wt% in the diffusion zone near the nitrided interface. The N map in Figure 6 also confirmed a sharp decrease in N content from the top surface to the nitride interface. Furthermore, the N content decreased gradually to a minute concentration with increasing distance from the nitrided interface. Moreover, a high O content of 8.15 wt% was found in the near-surface zone, and a sharp drop in its concentration to 0.57 wt% at the site 8 μm away from the top surface was found. The O concentration was relatively low in the diffusion zone,, which was also confirmed by the O map shown in Figure 6. It was noticed that the C content was high at the site 8 μm from the top surface. It was implied that C and N would co-diffuse into the 4140 steel during nitro-carburizing in the salt bath.

### 3.3. Hardness Profile of the QPQ-Treated Sample

**Figure 7:**
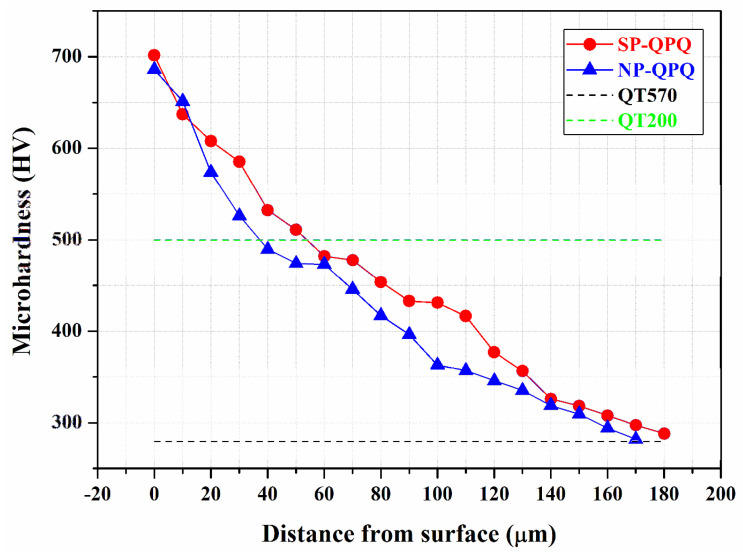
The micro-Vickers hardness profile of the NP-QPQ and SP-QPQ samples from the outermost layer to the interior.

Figure 7 demonstrates the microhardness distribution of the examined sample from the external surface to the interior of the QPQ-treated sample. Moreover, the hardness of the 4140 steel, which was solutionized and then tempered either at 200 °C (QT200) or 570 °C (QT570), is included in Figure 7. The QT200 sample, which was tempered at a low temperature, had a high hardness of HV 500. The hardening depth was defined from the top surface to a specific site, where the hardness was 50 HV_0.3_ higher than the core hardness. As shown in Figure 6, the defective nitrided layer near the outer surface caused a fluctuation in micro-hardness measurement. The results indicated that the hardness profile showed a gradual drop in hardness from the external surface of HV 700 to the interior of HV 270 at a depth of about 160 μm for both QPQ-treated samples. Compared with the core hardness, the nitro-carburizing treatment could markedly increase the surface hardness of the treated 4140 steel. Moreover, within a 120 μm depth from the top surface, the SP-QPQ sample possessed a slightly higher hardness than the NP-QPQ one at the same site.

### 3.4. EBSD Analysis

**Figure 8:**
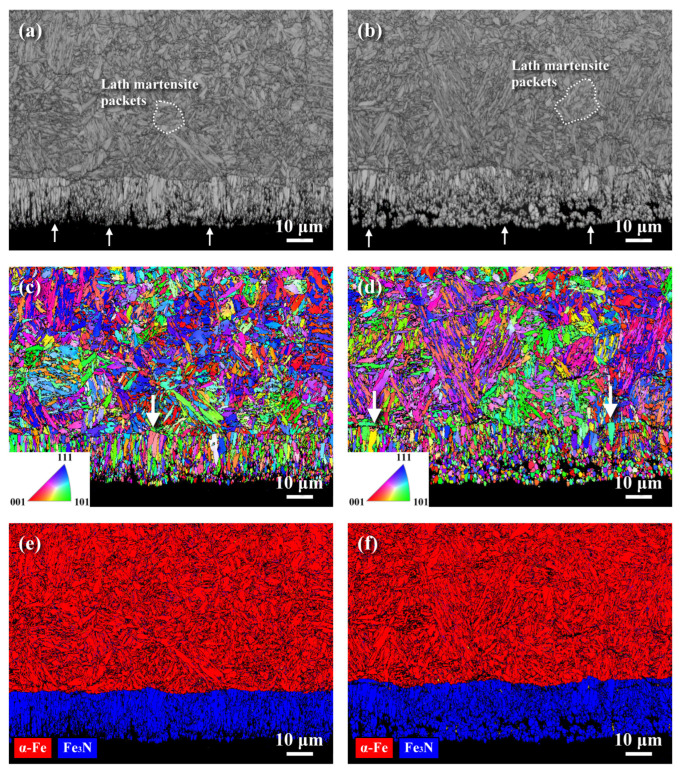
EBSD analysis of the QPQ-treated samples in cross-sectional view: (**a**,**b**) the band contrast (BC) maps, (**c**,**d**) the inverse pole figures (IPF), and (**e**,**f**) the phase maps. (**a**,**c**,**e**) The NP-QPQ sample. (**b**,**d**,**f**) The SP-QPQ sample.

The EBSD analysis displaying the surface microstructures in a cross-sectional view of the QPQ-treated samples is shown in Figure 8. The band contrast (BC) displayed the microstructure; in addition, an inverse pole figure (IPF) map and phase map were used to identify the grain sizes as well as the phases present in distinct zones of the inspected sample; after being solution-quenched and tempered at 570 °C for 2 h, lath martensite packets in different orientations were found to be distributed in the 4140 matrix. Extremely fine grains were formed in the outermost zone of the QPQ-treated specimens (Figure 8a,b), regardless of the MSP. In addition, fine columnar grains were observed to be aligned in the direction normal to the interface between the compound layer and diffusion zone (Figure 8c,d). As revealed in prior work, MSP forms nano-grains in the peened zone of the 4140 steel [28]. After QPQ treatment, only a few refined grains were left at the bottom of the compound layer of the SP-QPQ specimen relative to the NP-QPQ one. It was deduced that the refined grains introduced by MSP were corroded during the nitro-carburizing in salt baths. Figure 8e,f are the phase maps of the QPQ-treated specimens. As displayed in Figure 8e,f, the compound layer consisted of predominantly Fe_2_N in a refined granular structure. Moreover, the SP-QPQ sample possessed a little thicker but finer Fe-nitrides than the NP-QPQ one, about 18 μm in the former and 21 μm in the latter. It seemed that the MSP could enhance the formation of a little thicker compound layer.

### 3.5. Residual Stress Measurements

**Figure 9:**
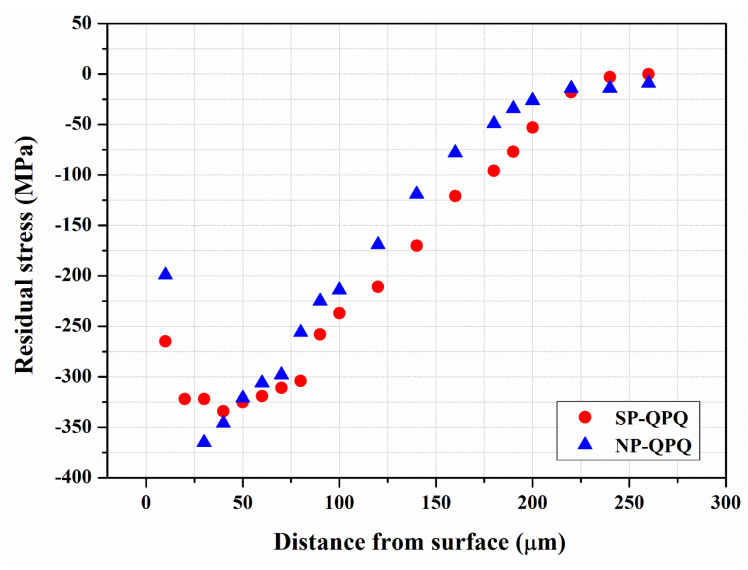
The residual stress distribution of the NP-QPQ and the SP-QPQ samples in the thickness direction from the surface to the interior with the 10 μm surface layer ground away before measurements.

Figure 9 displays the typical residual stress at distinct zones from the external surface to the interior of the examined samples and the calculated values in distinct depths of the samples. A high fluctuation in measured residual stress was obtained with the oxide and surface defect on the top surface of the investigated samples. To avoid interference from the oxide film and surface defect, about 10 μm in thickness of the investigated sample was ground off by sandpaper before stress measurement. The QPQ-treated sample consisted of different zones at different depths. Therefore, the stress measurement should relate to the significant phase at the specific site. Near the surface zone within the compound layer, the stress measurement was calculated using the XRD pattern around a 2θ angle of 135.5°, which was associated with the diffraction of Fe_2_N. At the depth above 20 μm from the external surface, the XRD pattern of α-Fe around a 2θ angle of 156.4° was detected and used to determine the residual stress at this site. It was known that hardened cases after nitro-carburizing and induced surface deformation by shot-peening were expected to introduce the CRS into the treated material [41]. The results indicated that the peak CRS was present in the subsurface layer and had a value of around –350 MPa. Overall, the two tested samples had a similar stress profile. As revealed in prior work, MSP could introduce high compressive stress into the severely peened zone but within a limited depth [22,28]. The associated tempering effect during nitro-carburizing in a salt bath at 570 °C/2 h had caused a stress relaxation, which MSP introduced. As a whole, a slightly wide compressive stress field was present in the SP-QPQ sample. The presence of a compressive stress field on the outer surface was expected to retard the fatigue crack initiation and growth of the QPQ-treated specimen.

### 3.6. Fatigue Tests

**Figure 10:**
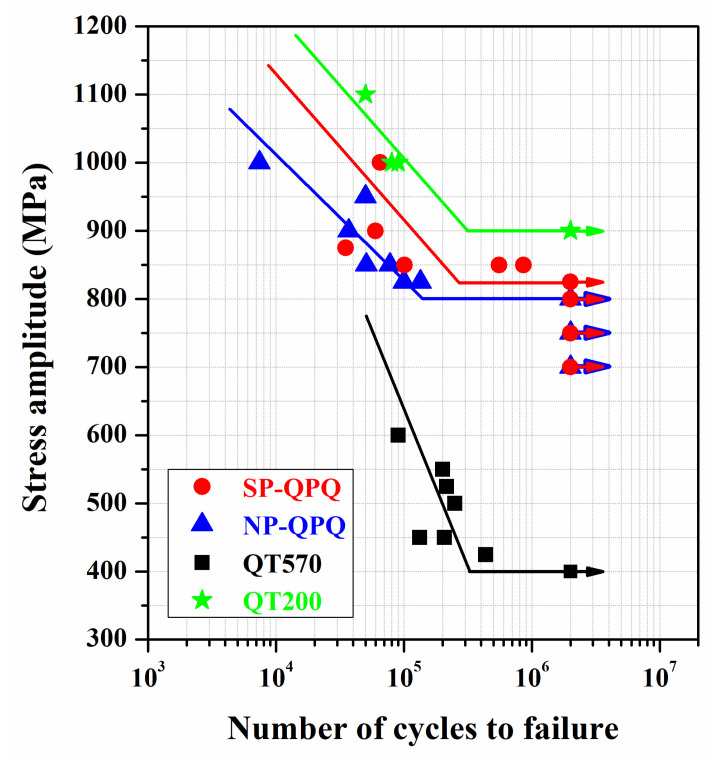
Fatigue stress (S) versus cycle (N) curves of the NP-QPQ and the SP-QPQ samples compared with the 4140 steel (QT200, QT570) samples.

The stress amplitude (S) versus cycle number (N) of the tested samples after rotating bending fatigue tests is shown in Figure 10. During nitro-carburizing, the 4140 substrate received a high-temperature tempering at 570 °C, which caused a decline in the substrate’s hardness to HV 270. The fatigue life of the 4140 substrate (QT570), which had been tempered at 570 °C/2 h, showed decreased cyclic stress with increased cyclic life. The fatigue limit of the 570 °C tempered substrate could be as low as 400 MPa. As revealed in a prior study, the fatigue limit of the QT200 sample [28] was about 875 MPa. Increasing the tempering temperature resulted in a significant decrease in the fatigue strength of the 4140 steel. In addition, both the QPQ-treated samples showed similar fatigue performance, regardless of the MSP. The NP-QPQ and SP-QPQ samples’ fatigue limits were about 800 and 825 MPa, respectively. The fatigue strength of the QPQ-treated samples was much greater than that of the 570 °C tempered substrate (QT570). With the nitrided layer, the QPQ-treated sample possessed a superior fatigue performance relative to the tempered substrate (QT570). Although the oxide and nitride layer coated on the external surface of the QPQ-treated samples was hard and brittle, it did not damage their fatigue properties as compared with the QT570 sample. Moreover, the SP-QPQ sample had a slightly better fatigue performance than the NSP-QPQ loading at the cyclic stress around the fatigue limit. In addition, the results indicated that the brittle compound and oxide layers on the QPQ-treated sample did not shorten its fatigue life or degrade its fatigue strength relative to the QT570 sample.

### 3.7. Fractured Surface Examinations

**Figure 11:**
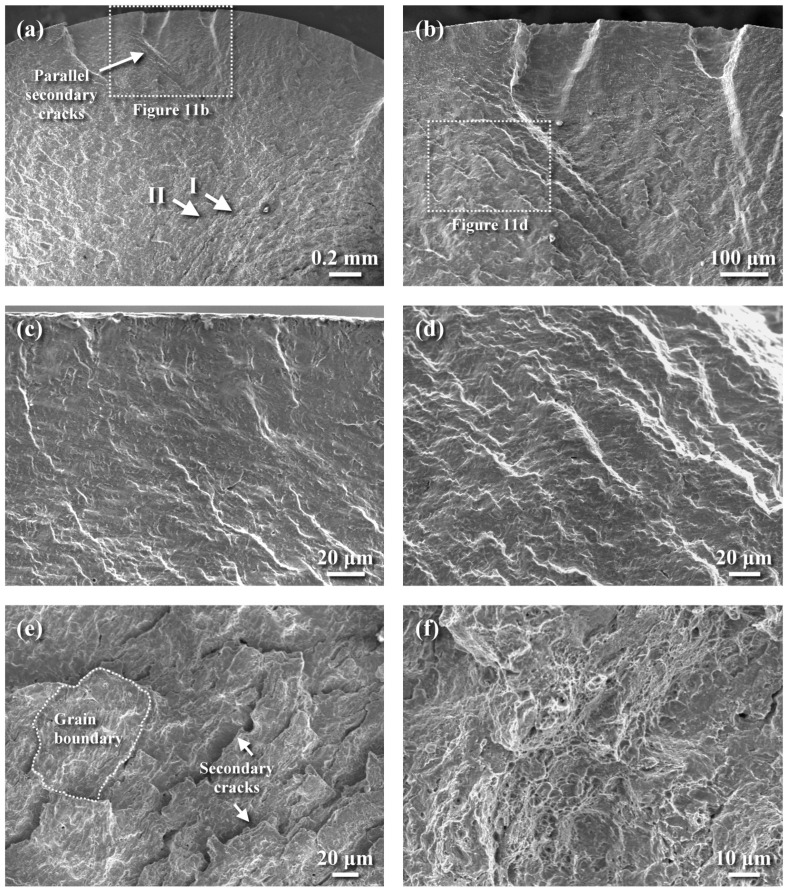
Fatigue-fractured appearance of the QT570 sample: (**a**) the macro-fractured appearance, (**b**) the enlarged view of the dashed zone in Figure 11a, (**c**) transgranular brittle fracture of the crack initiation site, (**d**) the enlarged view of the dashed zone in Figure 11b showing quasi-cleavage with tearing separation, (**e**) the enlarged view of the site indicated by the arrow II in Figure 11a showing quasi-cleavage with grain boundary cracking, and (**f**) the ductile dimple rupture of the final fracture zone.

The fatigue-fractured feature of the QT570 sample is shown in Figure 11. The induced peak tensile stress on the sample’s surface during the rotating bending fatigue test was expected to cause crack initiation on the outer surface and propagate inward. It was found that multiple cracks were initiated at the external periphery of the fatigue-fractured QT570 sample (Figure 11a,b). As the crack grew inwards, a smooth transgranular appearance with an increased morphology of the tearing ridge was observed, as shown in Figure 11b,c. Examining at higher magnification (Figure 11d), a quasi-cleavage-like fracture mixed with tear separation occurred in this zone. A transition zone with parallel secondary cracks was present between the relatively flat fracture region and rough final fracture zone, as indicated by arrow I in Figure 11a. Figure 11e shows a quasi-cleavage fracture accompanied by grain boundary cracking, as indicated by arrow II in Figure 11a. In addition, fine lamellar separations were more likely to be seen in the region before the final fracture. The change in fracture mode was not known at this moment. As the crack propagated further into the central portion, a fine dimple rupture appeared in the final fracture zone (Figure 11f), which was associated with the ductile nature of the 4140 steel tempered at high temperature.

**Figure 12:**
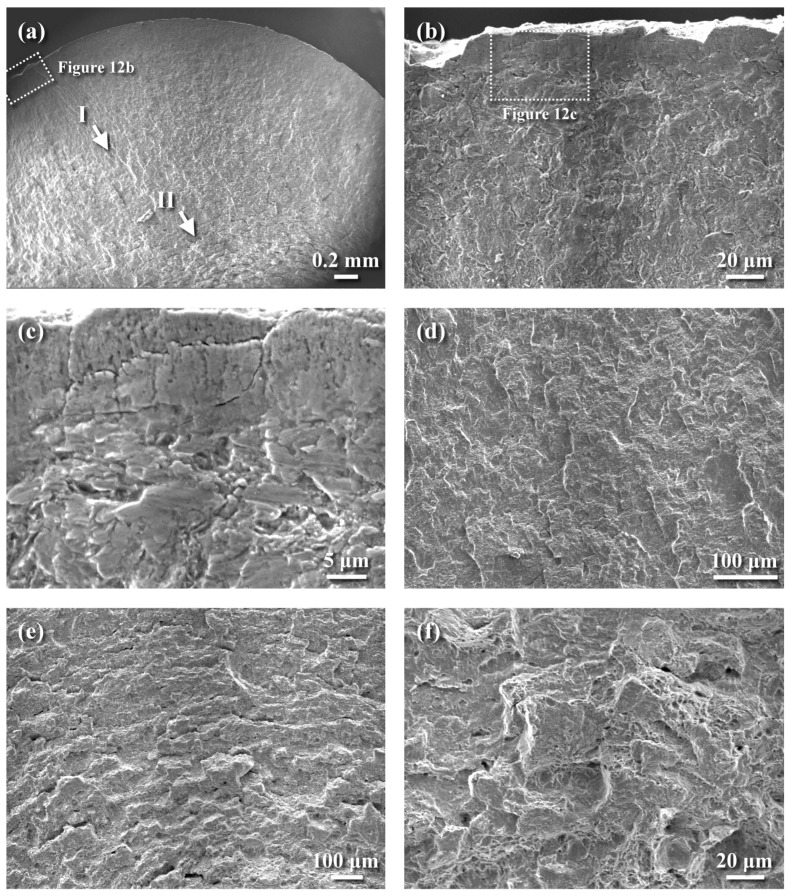
SEM photographs showing the fatigue-fractured features of the QPQ-treated sample under a stress of 850 MPa: (**a**) macro-fractured appearance, (**b**) the cracking and spalling of the top surface zone, (**c**) the enlarged view of the dashed zone in (**b**) showing the rubbed surface feature of the outer surface zone, (**d**) quasi-cleavage fracture with tear separation at the site indicated by arrow I in (**a**), (**e**) a change in fracture mode before final rupture, indicated by arrow II in (**a**), and (**f**) quasi-cleavage fracture mixed with ductile dimple in the transition zone.

The typical fracture appearance of the NP-QPQ-treated sample under a stress of 850 MPa after the fatigue test, regardless of MSP, is shown in Figure 12. The macro-view displayed a skinny, brittle layer decorated on the outer profile of the examined specimen (Figure 12a,b). Cracking and spalling were more likely to occur in the outermost hardening case (Figure 12b), implying the ease of fatigue initiation. It was noticed that a rubbed feature (Figure 12b), not a cleavage-like fracture of the compound layer, was seen in the outer surface zone. Such fracture features should be related to the CRS field, inducing a crack-closure effect. Inspecting the surface zone at higher magnification (Figure 12c), the compound layer with lamellar cracks normal to the outer surface could be associated with the strike-like oxide film dispersed in the compound layer. In addition, the diffusion zone beneath the compound layer also exhibited a feature similar to that of the compound layer, but without the lamellar texture present in the latter (Figure 12c). As the crack extended about 0.65 mm away from the surface, a quasi-cleavage fracture with tear separation implied an increased crack growth rate (Figure 12d), as indicated by arrow I in Figure 12a. Similar to the QT570 sample, a marked transition in fracture appearance occurred in the region about 1.8 mm from the external surface of the inspected sample (Figure 12e). Deep secondary cracks were more likely to be observed in this zone, as indicated by arrow II in Figure 12a. A change in fracture morphology occurred from a quasi-cleavage fracture to quasi-cleavage mixed with dimple ahead of the final fracture zone (Figure 12f).

**Figure 13:**
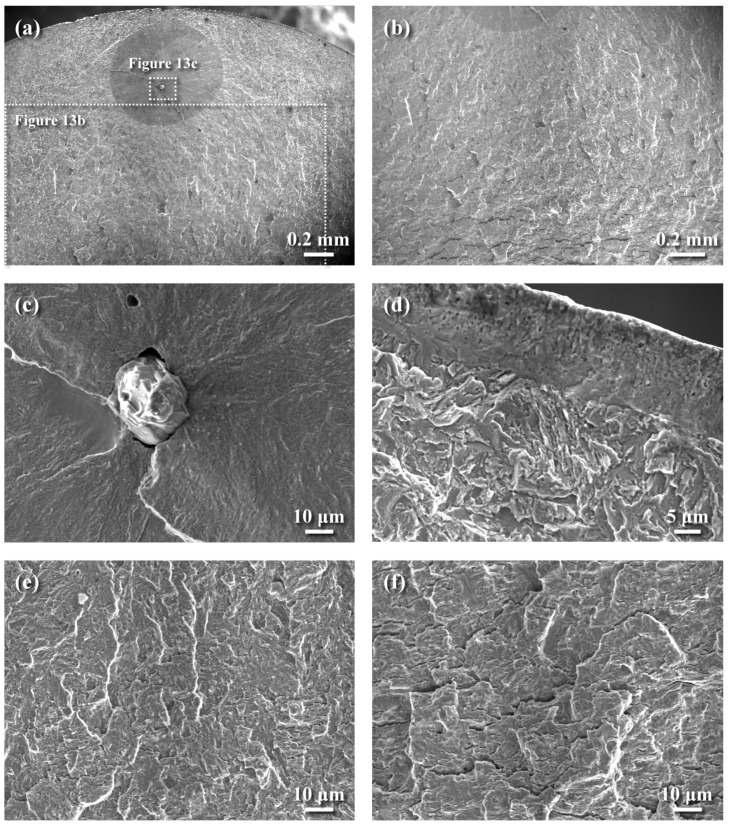
The fatigue-fractured morphologies of the SP-QPQ sample tested under a stress of 850 MPa: (**a**) macro-fractured appearance, (**b**) a change in fracture morphology from the fish-eye zone to the fracture zone with deep secondary cracks, (**c**) subsurface crack initiation at the inclusion, (**d**) the rubbed surface feature of the compound layer, (**e**) fatigue-fractured feature in the region outside the fish-eye zone, and (**f**) quasi-cleavage-like fracture with lamellar separations normal to the crack growth direction.

Figure 13 shows the fatigue-fractured morphologies in distinct zones of the SP-QPQ sample tested under a stress of 850 MPa. The macro-fractured appearance displayed the presence of the fish-eye zone in the inspected sample (Figure 13a), which was responsible for the fatigue crack initiation in the subsurface zone. The subsurface crack was expected to grow outwards and inwards simultaneously until the final fracture occurred. A gradual change in fracture morphologies from the fish-eye zone to the final fracture zone was investigated in distinct sites (Figure 13b). Figure 13c shows the fatigue crack initiated at the subsurface inclusion with high Al, Ca, and O concentrations, which was identified by EDS analysis. In prior work, the subsurface Ca aluminate identified by a JXA-8200 electron probe microanalysis (EPMA, JEOL, Tokyo, Japan) was responsible for the fatigue fracture of the gas-nitrided 4140 steel [28]. Moreover, the fish-eye zone exhibited a cleavage-like fracture appearance (Figure 13c). Examining the fracture appearance of the near-surface zone at higher magnification (Figure 13d), a rubbed compound layer with a trace of nitrided microstructure was found. The cracking and spalling of the outer surface layer were less likely to be inspected in the examined specimen. It was noted that the diffusion zones beneath the compound layer (Figure 13d) displayed the outline of prior austenite grain boundaries and revealed the martensite packets with fine precipitates at the lath boundaries within an austenite grain. On the opposite side of the fish-eye zone, transgranular fatigue showing the feature of quasi-cleavage was inspected (Figure 13e). As the crack propagated further into the sample (about 0.7 mm from the edge of the fish-eye zone), a predominant quasi-cleavage fracture with lamellar secondary cracks normal to crack growth direction was formed (Figure 13f). The fracture appearance of the inspected sample suggested that the fatigue crack would extend faster into the central part of the QPQ-treated sample than it would grow outwards to the external surface before the final fracture.

## 4. Discussion

After QPQ treatment, a compound layer with few fine pores was formed on the sample surface, regardless of shot-peening. EDS was applied to show the O and N distributions in the compound layer of the QPQ-treated specimens (Figure 6). The results indicated that O (Figure 6) was more likely to concentrate in the outermost layer, forming a continuous oxide film. By contrast, N (Figure 6) was rich in, and deep into, the compound layer. A strike-like oxide film was formed in the zone deep into the compound layer. The XRD pattern revealed that Fe_3_N mixed with a small amount of Fe_3_O_4_ was found around the top surface of the sample after salt-bath nitro-carburizing. In addition, the post-oxidized sample resulted in Fe_2_N instead of Fe_3_N, together with an increased amount of Fe_3_O_4_. It was deduced that the decomposition of the outmost nitrided layer during post-oxidation caused some N atoms to diffuse inwards. Thus, the extra N resulted in the transformation of Fe_3_N to Fe_2_N. Moreover, the refined nitrides assisted the O diffusion along the grain boundaries, forming a strike-like oxide film in the lower portion of the compound layer. In this study, MSP only caused a slight increase in the hardened depth (Figure 7) and compound layer thickness (Figure 8). It was found that few refined grains remained around the interface between the compound layer and diffusion zone of the SP-QPQ sample. Some refined grains introduced by MSP seemed to be corroded away during nitro-carburizing in a salt bath. Moreover, the IPF maps (Figure 8c,d) displayed limited grain size differences between the NP-QPQ and SP-QPQ samples. Therefore, MSP was expected to have little effect on improving the mechanical properties of the QPQ-treated samples. In this work, both the QPQ-treated samples, regardless of the MSP, showed similar fatigue performance. The fatigue limits of the NP-QPQ and SP-QPQ samples were about 800 and 825 MPa, respectively. In prior work [22], the peening intensity before surface modification plays an important role to improve the fatigue performance of the peened sample. High peening intensity causes an increase in the surface roughness, resulting in a degradation of the fatigue resistance of the peened 7075 Al in the as-peened condition [22]. However, pickling before anodizing partly removed the surface layer of the peened zone; therefore, the highly peened 7075 Al alloy had a higher fatigue strength than the less peened one after anodizing [22]. To further increase the fatigue strength/life of the QPQ-treated 4140 steel, increasing peening intensity before nitro-carburizing 4140 steel is an option.

As reported in the open literature [26,42,43,44], the combination of a hardened case and RCS field after nitriding causes an improvement in fatigue properties, especially by increasing case depth. A nitrided case retards the crack initiation and growth at the surface of 4140 steel [45]. Thus, subsurface crack initiation at the inclusions is reported to dominate the fatigue failure of the nitrided 4140 steel [25,42,46]. It is pointed out that the nitro-carburizing of medium C steel in a salt bath followed by post-oxidation in the nitrite-nitrate bath can increase its fatigue strength compared to untreated medium C steel [33]. Moreover, nitro-carburized medium C steel’s fatigue crack initiation site in a salt bath changes depending on the sample’s preparation conditions. With and without the oxide layer, the crack initiation site is located at the subsurface compound layer [33]. Removing the sample’s oxide and compound layer, the fatigue fracture will be from the surface of the nitro-carburized medium C steel in a very high-cycle regime [33].

In the case of nitro-carburized and post-oxidized 4135 steel, the fatigue crack initiates from the internal inclusion in a high-cycle fatigue regime [34]. Although microcracks initiate in the surface of salt-bath nitro-carburized 13Cr-4Ni steel, the fatigue strength/life of the nitro-carburized 13Cr-4Ni steel is still much greater than that of the untreated steel [30]. Therefore, the presence of the nitrided layer is beneficial for increasing the fatigue resistance of the treated steel relative to the untreated one, regardless of the fatigue initiation site. In this work, the cracking and spalling of the superficial compound layer resulted in the fatigue crack initiation and growth of all of the QPQ-treated samples fatigue-loaded at/above 850 MPa. The S–N curves showed that the fatigue strength/life of the QPQ-treated samples was much greater than that of the QT570 sample, in which all samples had the same interior hardness. It was obvious that the QPQ treatment was effective for improving the fatigue performance of 4140 steel. With the presence of an RCS field to induce crack closure, the brittle compound layer displayed a rubbed feature (Figure 12b,c), not a cleavage-like fracture, in the QPQ-treated samples. In addition, the compound layer with some lamellar cracks normal to the outer surface could be associated with the strike-like oxide film inter-dispersed in the compound layer (Figure 12c). It was obvious that the combination of the CRS field and hard case would hinder the dislocation motions at the sample surface, resulting in increasing the fatigue strength/life of the QPQ-treated samples.

In the case of a surface-hardened steel, cracks can initiate and grow at the surface or subsurface zone, depending on the test loading. With a combination of an ample case depth and CRS field, the crack initiation site, leading to fatigue failure, can be shifted from the external surface to the subsurface zone, particularly at the inclusions. Under relatively low-stress conditions, the compound layer present in the SP-QPQ sample did not act as the crack initiation zone. Fatigue crack initiation was more likely to occur at the subsurface inclusions in the SP-QPQ sample fatigue-loading at 850 MPa or a little above the fatigue limit (825 MPa). A stair-case or stepwise S–N curve for the QPQ-treated sample was not present in this work because the changes in crack initiation site occurred at a relatively low stress or near the fatigue limit (Figure 10). It is reported that the maximum size of the inclusion and the depth to the outer surface play an important role in the rotating bending fatigue life of bearing steel compared with the inclusion type [47]. The subsurface crack initiated at the Ca aluminate inclusion would grow outwards and inwards simultaneously before the final fracture of the SP-QPQ sample (Figure 13a). The fish-eye zone (Figure 13c) exhibited a cleavage-like fracture appearance; however, the rubbed compound layer showed a trace of its microstructure (Figure 13d). In addition, the diffusion zones beneath the compound layer displayed martensite packets with fine precipitates at the lath boundaries within the prior austenite grains (Figure 13d). The macro-fractured appearance of all of the tested samples showed that a transition zone with a relatively rough fracture feature was present before the final fracture region. A predominant quasi-cleavage-like fracture with lamellar secondary cracks normal to crack growth direction was found in the transition zone (Figure 11e). Obviously, the fatigue growth rate of the subsurface crack would be much faster toward the interior than toward the outer surface, which could be attributed to the combined act of the CRS and hard case in the SP-QPQ specimen. By contrast, multiple cracks were initiated at the external periphery and propagated inwards for the fatigue-fractured QT570 sample. It seemed that the ease of slip on the external surface of the QT570 sample was responsible for its fatigue limit of about 400 MPa relative to that of about 825 MPa of the QPQ-treated samples. Although the trend of the fatigue performance of QPQ-treated 4140 steel was disclosed, the cycle numbers of those tests in this work did not fall into the high-cycle fatigue regime. Further decreasing the fatigue-loading, the fatigue crack was expected to initiate at the subsurface zone for all of the QPQ-treated samples.

## 5. Conclusions

1. After QPQ treatment, O was more likely to concentrate in the outermost layer, forming a continuous oxide film, whereas N was enriched in the overall compound layer. In addition, O diffused inwards and assisted in forming a strike-like oxide film inter-dispersed in the compound layer. After salt-bath nitrocarburizing, Fe_3_N mixed with a small amount of Fe_3_O_4_ was found on the top surface of the treated sample. Moreover, Fe_2_N instead of Fe_3_N and an increased amount of Fe_3_O_4_ were found in the post-oxidized sample.
2. A gradual drop in hardness from the external surface of HV 700 to the interior of HV 270 at a depth of about 160 μm was obtained for both QPQ-treated samples. Moreover, MSP only caused a slight increase in the hardened depth and compound layer thickness after QPQ treatment. It could be that the associated tempering effect during nitro-carburizing in a salt bath at 570 °C/2 hrs had caused a relaxation of RCS introduced by MSP. Therefore, the SP-QPQ and NP-QPQ samples had similar residual stress profiles.
3. Cracking and spalling the superficial compound layer resulted in the fatigue crack initiation and propagation of all of the QPQ-treated samples fatigue-loaded at/above 875 MPa. The CRS field and hard case in the QPQ-treated specimen hindered dislocation motions around the surface, thereby increasing its fatigue resistance. Fatigue crack initiation was more likely to occur at the subsurface inclusions in the SP-QPQ sample fatigue-loading at 850 MPa or slightly above the fatigue limit (825 MPa). By contrast, multiple cracks were initiated at the external periphery and propagated inwards for the fatigue-fractured QT570 sample. The ease of slip on the external surface of the QT570 sample was responsible for its fatigue limit of about 400 MPa relative to that of about 825 MPa of the QPQ-treated samples.

## Figures and Tables

**Table 1:** The surface roughness of distinct samples.

Sample	Surface Roughness (μm)		
	Sa	Sp	Sv
QT200 [28]	0.31	2.02	1.75
QT200 + SP [28]	0.50	2.46	2.35
NP-QPQ	0.28	2.42	1.31
SP-QPQ	0.60	4.08	2.48

Note: Sa: arithmetical mean height of the surface; Sp: maximum peak height of the surface; and Sv: maximum pit depth of the surface.

**Table 2:** The chemical compositions in distinct zones of the SP-QPQ-treated sample.

Location	Distance from Surface (μm)	Chemical Composition (wt.%)							
		C	N	O	Mn	Cr	Mo	Si	Fe
Compound layer	2	0.45	5.21	8.15	0.41	0.91	0.08	0.25	Bal.
	8	1.30	5.45	0.57	0.78	0.88	0.10	0.19	Bal.
Diffusion zone	20	0.72	0.55	0.08	1.01	1.06	0.13	0.18	Bal.
	50	0.43	0.38	0.04	0.61	0.87	0.16	0.22	Bal.
Base metal	150	0.38	0.24	0.04	0.98	1.08	0.15	0.21	Bal.
	250	0.44	―	―	0.82	1.00	0.14	0.21	Bal.

## Data Availability

Dataset available on request from the authors.

## Abbreviations

BC	Band contrast
CRS	Compressive residual stress
EBSD	Electron backscatter diffraction
EDS	Energy-dispersive X-ray spectroscopy
EPMA	Electron probe microanalysis
FWHM	Full width at half maximum
IPF	Inverse pole figure
MSP	Micro-shot peening
PM	Phase map
QT200	4140 tempered at 200 °C
QT570	4140 tempered at 570 °C
SEM	Scanning electron microscope
XRD	X-ray diffractometer

## References

[1] Sirin Y.S., Kaluc E. (2012). Structural surface characterization of ion nitrided AISI 4340 steel. Mater. Des..

[2] She D., Yue W., Fu Z., Gu Y., Wang C., Liu J. (2013). The Effect of Nitriding Temperature on Hardness and Microstructure of Die Steel Pre-Treated by Ultrasonic Cold Forging Technology. Mater. Des..

[3] Sim A., Park C., Kang N., Kim Y., Chun E.-J. (2019). Effect of Laser-Assisted Nitriding with a High-Power Diode Laser on Surface Hardening of Aluminum-Containing Martensitic Steel. Opt. Laser Technol..

[4] Shen H., Wang L. (2020). Mechanism and Properties of Plasma Nitriding AISI 420 Stainless Steel at Low Temperature and Anodic (Ground) Potential. Surf. Coat. Technol..

[5] Ohtsu N., Endo R., Takeda S., Sakuraba Y., Hirano M. (2024). Open-Atmosphere Laser Nitriding of Austenitic Steels to Form Wear-Resistant Surfaces. Surf. Coat. Technol..

[6] Yeung C., Lau K., Li H., Luo D. (1997). Advanced QPC complex salt bath heat treatment. J. Mater. Process. Technol..

[7] Li H., Luo D., Yeung C., Lau K. (1997). Microstructural studies of QPQ complex salt bath heat-treated steels. J. Mater. Process. Technol..

[8] Marušić K., Otmačić H., Landek D., Cajner F., Stupnišek-Lisac E. (2006). Modification of carbon steel surface by the Tenifer^®^ process of nitrocarburizing and post-oxidation. Surf. Coat. Technol..

[9] Jacquet P., Coudert J., Lourdin P. (2011). How different steel grades react to a salt bath nitrocarburizing and post-oxidation process: Influence of alloying elements. Surf. Coat. Technol..

[10] Cai W., Meng F., Gao X., Hu J. (2012). Effect of QPQ Nitriding Time on Wear and Corrosion Behavior of 45 Carbon Steel. Appl. Surf. Sci..

[11] Min Y., Jiao X., Shao L., Chen Z., Jiang Z. (2023). Microstructure Characteristics of the Compound Layer Cracked during Grinding on QPQ-Treated Cr-Containing Steel Shafts. Adv. Eng. Mater..

[12] Campagnolo A., Dabalà M., Meneghetti G. (2019). Effect of Salt Bath Nitrocarburizing and Post-Oxidation on Static and Fatigue Behaviours of a Construction Steel. Metals.

[13] Xiong G.Y., He B.L., Zou R. (2008). Research of Microstructure and Properties of the 4Cr14Ni14W2Mo Steel with QPQ Salt-Bath Nitriding. Key Eng. Mater..

[14] Li G.-J., Peng Q., Wang J., Li C., Wang Y., Gao J., Chen S.-Y., Shen B.-L. (2008). Surface Microstructure of 316L Austenitic Stainless Steel by the Salt Bath Nitrocarburizing and Post-Oxidation Process Known as QPQ. Surf. Coat. Technol..

[15] Bonow V.T., Maciel D.S., Fenner N.L., Reguly A., Zimmer A., Zimmer C.G. (2021). Nitriding in Non-toxic Salts Bath: An Approach to Implement Cleaner Production in the Metallurgic Industry. Clean. Eng. Technol..

[16] Klemenz M., Schulze V., Rohr I., Löhe D. (2009). Application of the FEM for the Prediction of the Surface Layer Characteristics after Shot Peening. J. Mater. Process. Technol..

[17] Abdulstaar M., Mhaede M., Wollmann M., Wagner L. (2014). Investigating the effects of bulk and surface severe plastic deformation on the fatigue, corrosion behaviour and corrosion fatigue of AA5083. Surf. Coat. Technol..

[18] Benedetti M., Fontanari V., Bandini M., Savio E. (2015). High- and very high-cycle plain fatigue resistance of shot peened high-strength aluminum alloys: The role of surface morphology. Int. J. Fatigue.

[19] Karimbaev R., Pyun Y.-S., Maleki E., Unal O., Amanov A. (2020). An improvement in fatigue behavior of AISI 4340 steel by shot peening and ultrasonic nanocrystal surface modification. Mater. Sci. Eng. A.

[20] Kameyama Y., Komotori J. (2009). Effect of Micro Ploughing during Fine Particle Peening Process on the Microstructure of Metallic Materials. J. Mater. Process. Technol..

[21] Morita T., Noda S., Kagaya C. (2014). Influences of Fine-Particle bombarding and conventional shot peening on surface properties of steel. Mater. Trans..

[22] Su C.-H., Chen T.-C., Ding Y.-S., Lu G.-X., Tsay L.-W. (2023). Effects of Micro-Shot peening on the fatigue strength of anodized 7075-T6 alloy. Materials.

[23] Su C.-H., Chen T.-C., Tsay L.-W. (2023). Improved fatigue strength of Cr-electroplated 7075-T6 Al alloy by micro-shot peening. Int. J. Fatigue.

[24] Limodin N., Verreman Y. (2006). Fatigue strength improvement of a 4140 steel by gas nitriding: Influence of notch severity. Mater. Sci. Eng. A.

[25] Weidner A., Lippmann T., Biermann H. (2017). Crack initiation in the very high cycle fatigue regime of nitrided 42CrMo4 steel. J. Mater. Res..

[26] Hiraoka Y., Ishida A. (2017). Effect of Compound Layer Thickness Composed of γ’-Fe_4_N on Rotated-Bending Fatigue Strength in Gas-Nitrided JIS-SCM435 Steel. Mater. Trans..

[27] Kikuchi S., Komotori J. (2015). Evaluation of the Gas Nitriding of Fine Grained AISI 4135 Steel Treated with Fine Particle Peening and Its Effect on the Tribological Properties. Mater. Trans..

[28] Lin G.-W., Chen T.-C., Hsu H.-H., Tsay L.-W. (2024). Synergetic Effects of Micro-Shot Peening and Gas Nitriding on the Fatigue Performance of AISI 4140 Steel. Surf. Coat. Technol..

[29] Terres M.A., Laalai N., Sidhom H. (2012). Effect of Nitriding and Shot-Peening on the Fatigue Behavior of 42CrMo4 Steel: Experimental Analysis and Predictive Approach. Mater. Des..

[30] Hernández-Rengifo E., Rodríguez S.A., Coronado J.J. (2021). Improving fatigue strength of hydromachinery 13Cr-4Ni CA6NM steel with nitriding and thermal spraying surface. Fatigue Fract. Eng. Mater. Struct..

[31] Lee I. (2004). Post-Oxidizing Treatments of the Compound Layer on the AISI 4135 Steel Produced by Plasma Nitrocarburizing. Surf. Coat. Technol..

[32] Peng T., Dai M., Cai W., Wei W., Wei K., Hu J. (2019). The Enhancement Effect of Salt Bath Preoxidation on Salt Bath Nitriding for AISI 1045 Steel. Appl. Surf. Sci..

[33] Zhang J., Lu L., Shiozawa K., Zhou W., Zhang W. (2011). Effects of nitrocarburizing on fatigue property of medium carbon steel in very high cycle regime. Mater. Sci. Eng. A.

[34] Zhang J., Lu L., Shiozawa K., Zhou W., Zhang W. (2011). Effect of nitrocarburizing and post-oxidation on fatigue behavior of 35CrMo alloy steel in very high cycle fatigue regime. Int. J. Fatigue.

[35] Bhavsar V., Gujar A., Manthani N., Patil N., Pawar V., Mishra M.K., Singh R. (2020). Influence of Nitrocarburizing and Post-oxidation on Surface Characteristics, Fatigue, and Corrosion Fatigue Behaviour of AISI 4330V Steel. Trans. Indian Inst. Met..

[36] Lu G.X., Chen T.C., Shiue R.K., Tsay L.W. (2025). Effects of Surface Modifications on Rotating Bending Fatigue of Ni-Al Bronze Alloy. Metals.

[37] Tanaka K. (2019). The cosα method for X-ray residual stress measurement using two-dimensional detector. Mech. Eng. Rev..

[38] Tanaka K. (2018). X-ray measurement of triaxial residual stress on machined surfaces by the cosα method using a two-dimensional detector. J. Appl. Crystallogr..

[39] Alcantar-Martínez L.M., Ruiz-Trabolsi P.A., Tadeo-Rosas R., Miranda-Hernández J.G., Terán-Méndez G., Velázquez J.C., Hernández-Sánchez E. (2023). Improving the Surface Properties of an API 5L Grade B Pipeline Steel by Applying the Boriding Process. Part I: Kinetics and Layer Characterization. Coatings.

[40] Alcantar-Martínez L.M., Ruiz-Trabolsi P.A., Tadeo-Rosas R., Miranda-Hernández J.G., Miranda-Hernández J.G., Hernández-Sánchez E. (2023). Improving the Surface Properties of an API 5L Grade B Pipeline Steel by Applying the Boriding Process. Part II: On the Changes in the Mechanical Properties. Coatings.

[41] Hassani-Gangaraj S.M., Moridi A., Guagliano M., Ghidini A., Boniaridi M. (2014). The effect of nitriding, severe shot peening and their combination on the fatigue behavior and micro-structure of a low alloy steel. Int. J. Fatigue.

[42] Genel K., Demirkol M., Çapa M. (2000). Effect of ion nitriding on fatigue behaviour of AISI 4140 steel. Mater. Sci. Eng. A.

[43] Ashrafizadeh F. (2003). Influence of plasma and gas nitriding on fatigue resistance of plain carbon (Ck45) steel. Surf. Coat. Technol..

[44] Sirin S.Y., Sirin K., Kaluc E. (2013). Influence of initial conditions on the mechanical behavior of ion nitride AISI 4340 steel. Mater. Sci. Eng. A.

[45] Limodin N., Verreman Y., Tarfa T.N. (2003). Axial fatigue of a gas-nitrided quenched and tempered AISI 4140 steel: Effect of nitriding depth. Fatigue Fract. Eng. Mater. Struct..

[46] Sirin S.Y., Sirin K., Kaluc E. (2008). Effect of the ion nitriding surface hardening process on fatigue behavior of AISI 4340 steel. Mater. Charact..

[47] Xu L., Zhan Z., Zhang S. (2024). Influence of Inclusion Parameter and Depth on the Rotating Bending Fatigue Behavior of Bearing Steel. Metals.

